# Trends in documenting and coding of child physical abuse in hospitalised children and adolescents in Germany: a nationwide administrative data analysis

**DOI:** 10.1007/s00431-026-07300-y

**Published:** 2026-08-03

**Authors:** Teresa Walter, Nora Bentzien, Oliver Berthold, Andreas Jud

**Affiliations:** 1 Department of Child and Adolescent Psychiatry/Psychotherapy, University Hospital Ulm, Steinhövelstr. 5, 89075 Ulm, Germany; 2 Competence Center Child Abuse and Neglect Com.Can, Ulm, Germany; 3 School of Social Work, Zurich University of Applied Sciences, Zurich, Switzerland; 4 DRK Kliniken Berlin | Westend, Berlin, Germany

**Keywords:** Child physical abuse, Hospitalisation, Coding, ICD-10-GM

## Abstract

Hospital administrative data are widely used to monitor child physical abuse (CPA), but coding is often inconsistent. We described the national frequency and distribution of CPA-related coding in German administrative data and assessed how ICD-10-GM indicators and procedural coding influence ascertainment and temporal trends. We analysed aggregated German hospital data from the InEK data browser (2019–2024, ages 0–17 years), comparing T74.1 with broader indicator sets (a validated algorithm adapted to ICD-10-GM and an extended German set) and capturing procedural safeguarding documentation (OPS 1–945.x). We summarised principal diagnoses among hospitalisations with CPA-related coding and quantified CPA-related coding among admissions with predefined abuse-relevant injuries (fractures, haematomas) in children aged 0–2 years, a group at increased risk of physical abuse. CPA-related diagnostic coding remained rare (< 0.5% of annual admissions excluding procedural codes) but increased from 2019 to 2024, driven mainly by assessment- and psychosocial-context indicators (particularly Z04.5 and Z65), whereas T74.1 was stable or declining. Several low-frequency codes showed pronounced year-to-year variability. OPS 1–945.x—a child-protection assessment of suspected endangerment rather than confirmed abuse—had the highest counts and increased, with a decline in 2022. CPA-related indicators were mainly associated with head injuries, most often traumatic subdural haemorrhage and concussion. Among children with abuse-relevant fractures and haematomas, explicit T74.1 coding occurred in only a small minority, whereas OPS 1–945.x was more frequent.

*Conclusion*: These findings highlight the need for standardised coding guidance, validation of broader code sets, and training on administrative documentation to improve the surveillance value of hospital data.

**Table:** 

**What is Known:** • *CPA is under-coded in hospital administrative data; T74.1 is rarely used.* • *Broader ICD/procedure-based definitions can increase case capture.* **What is New:** • *German national discharge data (2019–2024) show stable/declining T74.1 with rising Z04.5/Z65 and increasing procedural codes OPS 1–945.x.* • *Including the broader indicator and procedural codes (e.g., Z04.5, Z65, OPS 1–945.x) reveals a marked shift in how concerns about CPA are documented, with these codes increasingly used alongside or instead of T74.1, particularly among children admitted with abuse-relevant injuries.*

**Supplementary Information:**

The online version contains supplementary material available at 10.1007/s00431-026-07300-y.

## Background

Child physical abuse (CPA) is a significant public health issue with severe long-term consequences [1]. Effective surveillance relies on consistent case identification and reporting to inform prevention and service planning [2]. Hospital discharge data are widely used for surveillance of public health conditions, as they capture diagnoses and procedures at scale over time. Given the key role of healthcare professionals in recognising and documenting CPA [3, 4], ICD-based administrative data have become essential for national monitoring, cross-country comparisons, and policy development [5–10]. However, validity is often compromised by coding practices leading to systematic underreporting [6, 9, 11–15]. E.g., the sensitivity of ICD-10-CM for CPA codes in the United States is estimated at 55.6% [13].

To improve CPA identification in hospital data, many international studies have applied broader algorithmic case definitions beyond explicit CPA codes [10, 16–21]. A validated French algorithm for children aged 1 month to 5 years showed good performance (positive predictive value, PPV, 86.3%) [22] and has been applied in comparative European analyses [10, 16].

In Germany, CPA can be documented within the G-DRG (Diagnosis Related Group) system using the ICD-10-GM code T74.1 (“physical abuse”). However, T74.1 is underused or recorded infrequently [14, 15]. This under-coding is attributed to various factors [14], including the reliance on broader indicator diagnosis codes (e.g., Z-codes). Additionally, procedural codes like OPS 1–945.x for child protection assessments are used more frequently to document CPA concerns [14, 15]. Consequently, CPA may be more reliably documented through injury diagnoses as well as broader indicator and procedural codes rather than through the explicit CPA code T74.1. Predefined abuse-relevant injuries can serve as an analytical window examining documentation pathways and sources of potential under-ascertainment.

In Germany, evidence remains limited on how using broader sets of diagnosis codes affects the identification and time trends of CPA-related coding in hospital data. Therefore, this study analyses national administrative hospital data from 2019 to 2024 to (i) describe the frequency and distribution of T74.1 and related diagnostic and procedural codes; (ii) examine how broader algorithmic code sets influence temporal trends; (iii) characterise the principal-diagnosis profiles of hospitalisations with CPA-related coding; and (iv) quantify CPA-related coding among young children admitted with predefined abuse-relevant injuries.

We use “CPA-related coding” as an umbrella term extending beyond T74.1 to broader maltreatment, assessment-related, external-cause, psychosocial-context and procedural safeguarding codes. These codes indicate administrative documentation of abuse-related diagnoses, not confirmed abuse; we therefore refer to “hospitalisations with CPA-related coding”, separating diagnostic and procedural categories.

## Methods

### Data source and study design

We conducted a retrospective, descriptive analysis of aggregated German inpatient discharge data from the publicly available data browser of the Institute for the Hospital Remuneration System (InEK). Under §21(3b) of the German Hospital Remuneration Act (KHEntgG), hospitals submit routine billing data for the G-DRG system, and the anonymised, aggregated outputs may be used for secondary analyses [23]. Coding is performed by physicians and/or clinical coding specialists. Because coding is reimbursement-driven, medico-legally sensitive or non-reimbursed codes such as T74.1 may be assigned selectively, whereas reimbursement-relevant procedural codes such as OPS 1–945.x may be recorded more systematically. The ICD-10-GM and OPS catalogues are revised annually; all codes used were available throughout 2019–2024, and OPS 1–945.0/1–945.1 have been reimbursement-relevant since 2018. Incremental revisions of coding guidance cannot be fully excluded.

### Study population and period

**Fig. 1:**
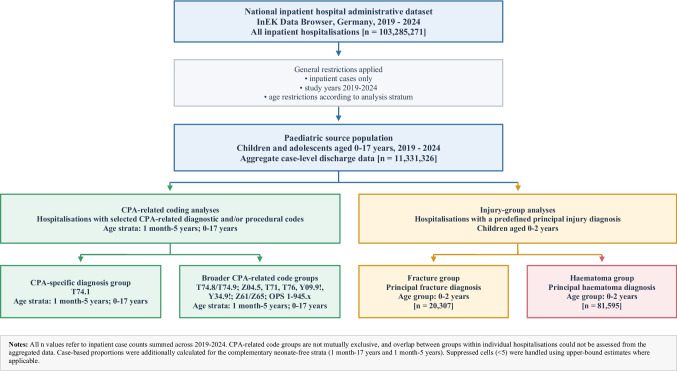
Derivation of analysis groups from the national inpatient dataset

The derivation of the analysis groups is shown in Fig. 1. The browser provides filtering from the 2019 data year onward. Inclusion criteria were inpatient status, age 0–17 years, and data year 2019–2024; exclusion criteria were outpatient/ambulatory cases, not captured by the §21 KHEntgG dataset. No person-level exclusions could be applied because only aggregated case-level data were available.

To assess coding frequency, counts of inpatient cases carrying the respective codes were related to inpatient admissions. Absolute counts and temporal trends are presented for the full 0–17 years population. Because this denominator includes many birth-related admissions that rarely carry CPA-related codes and inflate it, we additionally report a neonate-free 1 month-17 years denominator; the 1 month-5 years stratum corresponds to the validated age range used in previous studies [10, 16, 22]. Absolute counts and trends are unaffected by birth-related admissions.

CPA-related codes and their principal diagnoses were examined in the 1 month-5 years and 0–17 years strata. Abuse-relevant injury-group analyses were restricted to children aged 0–2 years [24]; admissions with the relevant injury were used as a denominator.

### Operational definition of code sets

We assessed (i) the CPA-specific ICD-10-GM diagnosis T74.1 and (ii) an extended German set (Table 1), derived from the validated French algorithm [16, 22] that was subsequently applied in comparative European analyses [10] and adapted to ICD-10-GM (2025) using prior German work and a systematic review [14, 15]. Codes were grouped into five categories: (1) the CPA-specific diagnosis T74.1; (2) broader maltreatment diagnoses (T74.8, T74.9); (3) assessment-, external-cause and injury-context indicators (Z04.5, T71, T76, Y09.9!, Y34.9!); (4) psychosocial-context diagnoses (Z61, Z65); and (5) the procedural safeguarding code OPS 1–945.x. In ICD-10-GM—unlike ICD-10-CM—T76 does not denote suspected maltreatment, and Z61 and Z65 are three-character categories whose abuse-relevant content is a non-separable inclusion term mixed with unrelated psychosocial circumstances. OPS 1–945.x was analysed separately as a procedure code. Codes unavailable in ICD-10-GM (W50, X91–X99, Y00–Y04, Y08, Y87.1) were excluded; the extended German set was not validated (see Limitations). An exclamation mark (e.g., Y09.9!) denotes a mandatory secondary code recorded together with a principal code. OPS 1–945.0/1–945.1 record a structured, multidisciplinary assessment in cases of suspected child endangerment, irrespective of whether maltreatment is confirmed; they therefore mark documented safeguarding assessment rather than confirmed abuse.

**Table 1:** ICD-10-GM and OPS codes used to define CPA-related coding in the extended German algorithm compared with the algorithm used in the European study [10]

Category	Code	Description	German algorithm	European algorithm
CPA-specific diagnosis	T74.1	Maltreatment syndromes – physical abuse	✓	✓
Broader maltreatment diagnoses	T74.8	Maltreatment syndromes – other maltreatment syndromes	✓	—
	T74.9	Maltreatment syndromes – maltreatment syndrome, unspecified	✓	—
Assessment, external-cause and injury-context indicators	Z04.5	Examination and observation following an alleged or inflicted injury	✓	✓
	T71	Asphyxiation	✓	✓
	T76	Unspecified effects of external causes	✓	—
	Y09.9!	Assault	✓	✓
	Y34.9!	Unspecified event, undetermined	✓	—
Psychosocial-context diagnoses	Z61	Problems related to childhood experiences (including suspected physical abuse)	✓	—
	Z65	Problems related to other psychosocial circumstances (including victim of crime and terrorism)	✓	✓ (Z65.4)
Procedural child-protection/safeguarding assessment – OPS	1–945.0	Diagnostic procedures in cases of suspected risk to a child’s wellbeing or health – without further measures	✓	—
	1–945.1	Diagnostic procedures in cases of suspected risk to a child’s wellbeing or health – with implementation of at least one formally documented case conference	✓	—
European algorithm codes not available in the German algorithm	W50	Hit, struck, kicked, twisted, bitten or scratched by another person	—	✓
	X91–X99 (excl. X96)	Assault by specified means	—	✓
	Y00–Y04	Assault by blunt object, by pushing, or by bodily force	—	✓
	Y08	Assault by other specified means	—	✓
	Y87.1	Sequelae of assault	—	✓

### Principal diagnoses by CPA-related codes

To describe the diagnostic profile, we extracted principal diagnoses for hospitalisations carrying the CPA-specific diagnosis, broader indicators or the procedural code. In the G-DRG system the principal diagnosis is the single diagnosis chiefly responsible for the admission. Where several relevant conditions co-exist, the others appear as secondary diagnoses.

We additionally defined two abuse-relevant injury groups by principal diagnosis—haematomas (superficial injuries of the ears, scalp, oral mucosa, buttocks, neck and genitals) and fractures (skull, ribs, humerus, clavicle, radius/ulna, femur and tibia/fibula) (Table S1). For each injury group, we separately queried the case-level co-occurrence of each CPA-related diagnostic code, and of OPS 1–945.x, with the relevant principal diagnosis. Because the browser provides only separate aggregate counts for each code, we could not determine whether multiple CPA-related codes occurred within the same hospitalisation; admissions carrying several codes are therefore counted once per code. Person-level identifiers were unavailable, so repeat admissions could not be identified.

As an exploratory analysis, we described predefined fracture diagnoses by anatomical site (0–2 years), reporting total admissions (2019–2024) and, where individually reportable, T74.1, Z04.5 and OPS 1–945.x. Because several site-by-code cells were suppressed, these are reportable minimum counts rather than exact site-specific abuse probabilities.

### Suppressed cells and upper-bound estimates

For data protection, the browser suppresses counts below five (shown as “ ≤ 4”, i.e., 1–4 cases). For the fracture and haematoma groups we calculated diagnosis-based coding proportions under two assumptions: each suppressed cell set to four (upper bound) and, as a sensitivity analysis, to one (Table S6). Both assume no overlap between diagnosis codes within the same case, as the browser reports each code only as a separate aggregate count; the “upper bound” is therefore the maximum diagnosis-based proportion under these assumptions. OPS 1–945.x was treated separately and excluded from these estimates.

### Statistical analysis

Aggregated outputs were analysed descriptively in Microsoft Excel. We summarised annual paediatric admissions by age group (1 month-5 years; 0–17 years; neonate-free 1 month-17 years) and identified CPA-related coding via T74.1, the extended indicator set and/or OPS 1–945.x. Because the data are aggregated at case level, counts refer to inpatient cases carrying the respective code, not distinct children; consequently, repeat admissions and within-case code overlap cannot be assessed. We report annual counts, proportions and the distribution of individual codes. Temporal trends were summarised with three metrics: net percent change (2019–2024); relative range [(maximum—minimum)/mean]; and Range/|Net| [from absolute annual counts: (maximum − minimum)/|2024–2019|, set to ∞ when equal], where values near 1 indicate a broadly directional trend and values > 1 indicate within-period fluctuation exceeding the net change. Hospitalisations with CPA-related coding were pooled across 2019–2024 for the most frequent principal diagnoses. For the fracture and haematoma groups (0–2 years) we report annual group sizes and proportions with CPA-related indicators and/or OPS 1–945.x. No inferential testing was performed.

## Results

### Sample characteristics

Annual inpatient cases averaged 529,834 in the 1 month–5 years stratum, 1,888,554 in the 0–17 years stratum, 1,105,162 in the 0–2 years stratum and 1,127,105 in the neonate-free 1 month–17 years stratum. Sex distribution was broadly balanced across groups, with a slight male predominance. Age composition differed, with the highest proportions in children aged 1–2 and 3–5 years in the 1 month-5 years group, and in neonates (< 28 days) in the 0–2 years (68.9%) and 0–17 years (40.3%) groups, reflecting the large contribution of birth-related admissions to these strata (Table 2).

**Table 2:** Sample Characteristics

	0–17 years	1 month-5 years	0–2 years
Mean annual cases	1,888,554	529,834	1,105,162
Sex, mean %			
Female	47.1	43.0	46.8
Male	52.9	57.0	53.2
Diverse	0	0	0
Unknown	< 0.1	< 0.1	< 0.1
Age, mean %			
< 28 days	40.3	-	68.9
< 1 year	8.4	30.1	14.4
1–2 years	9.8	34.8	16.7
3–5 years	9.9	35.1	-
6–9 years	9.0	-	-
10–15 years	15.3	-	-
16–17 years	7.3	-	-

### Temporal trends in CPA-related coding

**Fig. 2:**
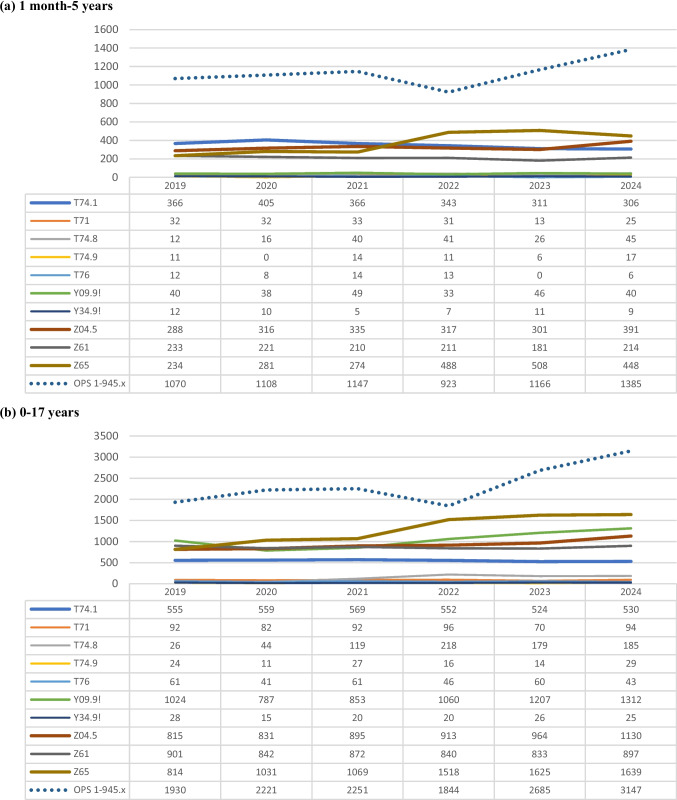
Temporal trends in CPA-related diagnostic and procedural coding. **a** Children aged 1 month-5 years; **b** children and adolescents aged 0–17 years

1 month-5 years. (Fig. 2a; Table 3; Figure S2). The CPA-specific code T74.1 declined from its 2020 peak to 306 cases in 2024 (−16.4% vs 2019). T74.8 increased, whereas several rare CPA-related codes showed pronounced year-to-year fluctuation and non-monotonic trajectories. In contrast, broader psychosocial-context and assessment indicators, particularly Z65 and Z04.5, increased more consistently, while Y09.9! remained broadly stable despite fluctuations. Overall, low-frequency codes were largely variability-dominated (Range/|Net|> 1), whereas for the broader indicator codes and the total diagnostic code volume, variability was more closely aligned with net change (Range/|Net| ≈ 1). Total diagnosis codes (excluding OPS) increased from 1,240 in 2019 to 1,501 in 2024 (+ 21.0%), corresponding to an average of 0.3% of inpatient cases; including OPS yielded an upper-bound average proportion of 0.5%.

**Table 3:** Descriptive metrics of temporal change and variability in CPA-related coding (2019–2024)

1 month-5 years					
Code	Net change (n)	Net % change (2019–2024)	Range (n)	Relative range (% of mean)	Range/|Net|*
T74.1	−60	−16.4%	99	28.3%	1.7
T71	−7	−21.9%	20	72.3%	2.9
T74.8	33	275.0%	33	110.0%	1.0
T74.9	6	54.5%	17	172.9%	2.8
T76	−6	−50.0%	14	158.5%	2.3
Y09.9!	0	0.0%	16	39.0%	∞
Y34.9!	−3	−25.0%	7	77.8%	2.3
Z04.5	103	35.8%	103	31.7%	1.0
Z61	−19	−8.2%	52	24.6%	2.7
Z65	214	91.5%	274	73.6%	1.3
OPS1-945.x	315	29.4%	462	40.8%	1.5
Total diag (excl. OPS)	261	21.0%	261	18.9%	1.0
0–17 years					
Code	Net change (n)	Net % change (2019–2024)	Range (n)	Relative range (% of mean)	Range/|Net|*
T74.1	−25	−4.5%	45	8.2%	1.8
T71	2	2.2%	26	29.7%	13.0
T74.8	159	611.5%	192	149.4%	1.2
T74.9	5	20.8%	18	89.3%	3.6
T76	−18	−29.5%	20	38.5%	1.1
Y09.9!	288	28.1%	525	50.5%	1.8
Y34.9!	−3	−10.7%	13	58.2%	4.3
Z04.5	315	38.7%	315	34.1%	1.0
Z61	−4	−0.4%	68	7.9%	17.0
Z65	825	101.4%	825	64.3%	1.0
OPS1-945.x	1217	63.1%	1303	55.5%	1.1
Total diag (excl. OPS)	1544	35.6%	1641	33.0%	1.1

*Range/|Net| is a ratio that compares the within-period variability (maximum − minimum) of a code count with the absolute net change between 2019 and 2024; values close to 1 indicate that the first and last observations are also the extreme values, consistent with a broadly directional trend, whereas values > 1 indicate that within-period fluctuation exceeds the net change, suggesting a less stable pattern. Net % change is normalised to the 2019 count, the relative range to the 2019–2024 mean; Range/|Net| is calculated from the absolute Net change (n) and Range (n) columns. It is set to ∞ when × 2024 =  × 2019

0–17 years (Fig. 2b; Table 3; Figure S3) T74.1 remained largely stable (−4.5% from 2019 to 2024), whereas T74.8 showed the strongest increase, albeit with substantial variability. Broader psychosocial-context and assessment indicators again increased, most clearly Z65 and Z04.5, while several low-frequency CPA-indicating codes showed pronounced fluctuation over time. As in younger children, variability exceeded net change for many rare codes (Range/|Net|> 1), whereas trends in Z65 and Z04.5 were more consistent with sustained increases (Range/|Net| ≈ 1). Total diagnosis codes (excluding OPS) rose from 4,340 in 2019 to 5,884 in 2024 (+ 35.6%), corresponding to an average of 0.3% of inpatient cases. OPS 1–945.x increased from 1,930 to 3,147 (+ 63.1%), despite a trough in 2022; including OPS yielded an upper-bound estimate of 0.4%. For the neonate-free 1 month–17 years stratum, which excludes birth-related admissions, the corresponding average proportions were 0.4% excluding OPS and an upper bound of 0.6% including OPS.

### Distribution of CPA-related codes

**Fig. 3:**
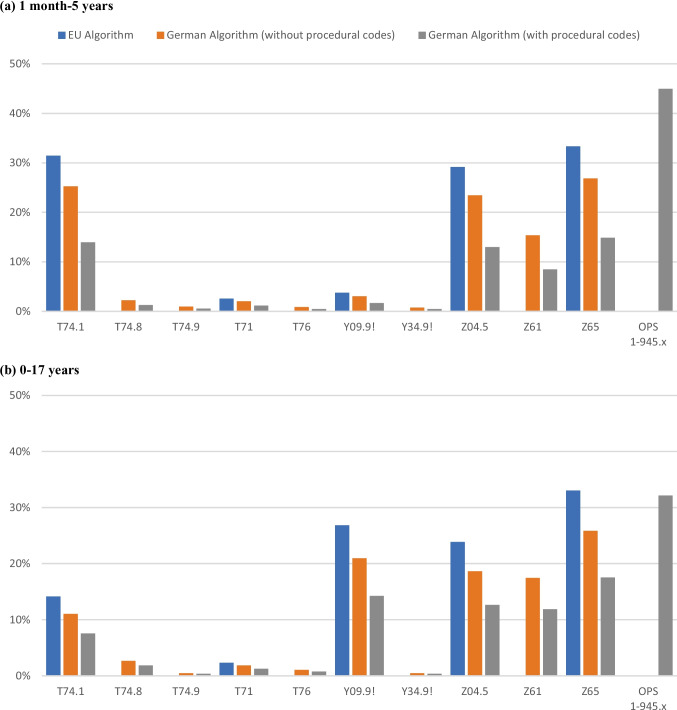
Distribution of CPA-related ICD-10-GM and OPS codes by age group and algorithm. **a** Children aged 1 month-5 years; **b** children and adolescents aged 0–17 years. *Note.* Categories are not mutually exclusive at the case level because a single hospitalisation may carry more than one code. Percentages represent the distribution of recorded code occurrences within each algorithm and should not be interpreted as proportions of independent case groups

1 month-5 years. In children aged 1 month-5 years (Fig. 3a), the EU algorithm concentrated on three ICD-10 indicators – T74.1 (31.4%), Z65 (33.3%) and Z04.5 (29.1%) – with all others contributing marginally (≤ 3.7%). The German algorithm (without procedural codes) showed a broader distribution, most frequently Z65 (26.8%), T74.1 (25.2%) and Z04.5 (23.4%), and a notable share of Z61 (15.3%). When procedural codes were included, OPS 1–945.x dominated (44.9%), accompanied by lower proportions of the ICD-10 indicators (e.g., T74.1 13.9%, Z65 14.8%, Z04.5 12.9%, Z61 8.4%).

0–17 years*.* In the 0–17 years group (Fig. 3b), coding shifted further towards psychosocial/external-cause indicators. For the EU algorithm, Z65 (33.0%), Y09.9! (26.8%) and Z04.5 (23.8%) predominated, while T74.1 contributed 14.1%. The German algorithm (without procedural codes) remained more heterogeneous, led by Z65 (25.8%), Y09.9! (20.9%), Z04.5 (18.6%) and Z61 (17.4%). With procedural codes included, OPS 1–945.x was the most frequent indicator (32.1%); among ICD-10 codes, Z65, Y09.9!, Z04.5 and Z61 remained most common.

### Principal diagnoses by code and age

Across the injury-related codes (T74.1, Z04.5, Y09.9!), principal diagnoses were dominated by head injuries in both age groups, particularly intracranial injury (S06) and superficial head contusion (S00) (Table S4). Under T74.1, traumatic subdural haemorrhage (S06.5) was the leading principal diagnosis in both strata (0–17 years: 8.7%; 1 month-5 years: 13.2%). For Z04.5 and Y09.9!, concussion (S06.0) predominated among 0–17-year-olds (19.1% and 42.0%, respectively), whereas in children aged 1 month-5 years superficial head contusion (S00.85) was most common, with concussion (S06.0) and subdural haemorrhage (S06.5) also frequent. In contrast, psychosocial-context codes (Z61, Z65) were more often associated with non-injury diagnoses, particularly developmental and behavioural disorders (e.g., F83); among the youngest children, Z65 was also linked to common paediatric and perinatal conditions (Table S4).

### Fractures and haematomas (ages 0–2)

Among admissions with abuse-relevant haematomas, 81,595 hospitalisations were identified between 2019 and 2024 (mean 13,599 per year; annual range 12,505–14,674) (Table S5). Explicit CPA coding with T74.1 remained uncommon (0.3–0.4% annually), while Z04.5 increased modestly from 0.1% in 2019 to 0.3% in 2024. OPS 1–945.x also increased over time, reaching 0.6% in 2024. Most other CPA-indicating ICD-10-GM diagnosis codes were absent or recorded only in very small numbers.

Among admissions with abuse-relevant fractures, 20,307 hospitalisations were identified between 2019 and 2024 (mean 3,385 per year; annual range 3,174–3,718). T74.1 ranged from 1.3% to 2.1% annually, while Z04.5 increased to 1.5% in 2024. OPS 1–945.x showed the clearest increase, reaching 5.8% in 2023 and remaining high in 2024 (5.2%). Other CPA-indicating ICD-10-GM diagnosis codes were absent or occurred only rarely, and Z61/Z65 remained uncommon.

Sensitivity analyses restricted to diagnosis codes showed that the handling of suppressed cells did not materially change the injury-group estimates (Table S6). Among fracture admissions, diagnosis-based CPA-related coding ranged from 2.3% to 3.9% when suppressed cells were set to one case and from 2.7% to 4.1% under the upper-bound assumption that each suppressed cell represented four cases. Among haematoma admissions, estimates ranged from 0.5% to 0.8% under both assumptions. OPS 1–945.x was analysed separately as procedural safeguarding documentation and remained more frequent than diagnosis-based CPA-related coding, particularly among fracture admissions.

In an exploratory fracture-site description (Table S7), skull/facial fractures were the largest subgroup (8,836 admissions, 2019–2024), followed by femur (4,223), radius/ulna (2,525), humerus (2,451), tibia/fibula (1,706), clavicle (474) and rib fractures (96). Reportable CPA-related coding varied by site, with the highest proportions in the small rib-fracture subgroup and higher reportable T74.1 and OPS 1–945.x for femur and humerus than for skull/facial or radius/ulna fractures; owing to small-cell suppression these are descriptive only.

## Discussion

This nationwide analysis of German inpatient administrative data yields five key findings. First, CPA-related diagnostic coding remained rare across all definitions, even as the overall volume increased. Second, this increase was driven not by T74.1 but by broader assessment-, external-cause-, psychosocial-context and procedural codes (Z04.5, Y09.9!, Z65, OPS 1–945.x). Third, low-frequency codes showed pronounced variability (Range/|Net|> 1), suggesting that trends reflect documentation behaviour rather than incidence changes. Fourth, the principal diagnoses of hospitalisations with CPA-related coding were dominated by head injuries, whereas the psychosocial-context codes Z61 and Z65 were more often linked to non-injury, developmental and behavioural diagnoses. Fifth, among young children with abuse-relevant fractures or haematomas, explicit CPA coding was uncommon whereas OPS 1–945.x was more frequent and increased. These data describe coding practices and cannot establish true CPA incidence, nor the accuracy of clinicians’ diagnostic decisions. The overall increase may reflect more frequent documentation but equally changes in coding behaviour, overlap between definitions or case mix, and given the small absolute numbers, relative changes should be interpreted cautiously. This is supported by the descriptive trend metrics and prior survey evidence of within-physician variability in documentation and reporting thresholds [25, 26]. Even with broader definitions, CPA-related diagnostic coding remained rare (< 1% of admissions). Although only partly comparable, other data sources underline this: A recent German household survey found that about 20.8% of respondents reported physical abuse in childhood [27]—a lifetime measure that cannot be equated with annual hospital coding. Only a subset of affected children present to hospital, and fewer still are admitted and receive CPA-related diagnostic or procedural coding. Child and youth welfare statistics, which use a different denominator, show that assessments identifying physical abuse in the context of acute or latent child endangerment rose from 15,063 in 2019 to 20,633 in 2024 [28]. Explicit hospital coding with T74.1 remained stable or declined, although the volume of broader diagnostic and procedural coding increased. Given the different populations, denominators and documentation pathways, these patterns should not be interpreted as evidence of clinical under-recognition. Rather, they indicate that hospital administrative coding captures only a small and selective part of the documented burden and that its surveillance value depends on coding and documentation practices rather than on clinical diagnostic performance.

The composition of CPA-related coding varied substantially by the code set applied. In younger children, the European algorithm yielded a profile in which T74.1 contributed similarly to broader indicators. This accords with comparative European evidence of marked heterogeneity across health systems, with some countries relying more on T74.1 and others on mechanism- or context-based indicators such as W50 and Z04.5 [10]. These findings highlight the lack of harmonised coding standards. Building on the multinational framework of Quantin et al. [10], harmonisation could be advanced by defining a core indicator set anchored in T74.1 with specified broader and procedural codes, reporting CPA-specific, broader diagnostic and procedural codes separately rather than as a single combined “CPA” measure, validating national algorithms against clinically adjudicated cases, and improving coding guidance to reduce documentation variability.

Interpretation of Z61 and Z65 requires particular caution. In ICD-10-GM these are three-character categories without codeable subdivisions, unlike the WHO subcodes Z65.4 and Z61.6, so their abuse-relevant content is a non-separable inclusion term mixed with unrelated psychosocial circumstances. Their increase more plausibly indicates greater documentation of psychosocial stressors than more confirmed abuse, and the lack of Z61.6/Z65.4 equivalents limits comparability with the European algorithm. The more frequent use of Y09.9! (“Assault”) in the 0–17-year age group may reflect interpersonal or peer violence, which might be more likely to be classified as assault than CPA.

A central system-specific feature aids interpretation: unlike ICD-10-CM, ICD-10-GM provides no dedicated code for suspected physical abuse, so suspected endangerment is documented primarily through OPS 1–945.x rather than diagnosis codes. Our findings are consistent with this, and imply that algorithms relying on ICD codes alone will under-capture suspicion-driven safeguarding activity in the German setting. However, higher OPS counts must not be read as more confirmed abuse: the code reflects suspicion-driven assessment including ruled-out cases and is reimbursement-relevant, so its increase more likely reflects training, for example by the German Society for Child Protection in Medicine (DGKiM) and implementation of structured child-protection pathways. The procedural and diagnostic indicators are therefore best interpreted separately.

The study period spans the COVID-19 pandemic, which complicates the interpretation of temporal trends. German national hospital discharge data show substantially reduced paediatric inpatient admissions in 2020–2022, indicating pandemic-related changes in admission volume, care-seeking behaviour and case mix [29]. Similarly, the multinational European study by Quantin et al. reported increased proportions of CPA hospitalisations in 2020 across several countries, interpreted mainly in the context of reduced overall hospital admissions during lockdown periods while CPA admissions remained broadly stable [10]. At the same time, increased parental stress and deterioration in family psychosocial health [30, 31] may have contributed to the rise in psychosocial-context codes, while pandemic-related disruption of hospital workflows may underlie irregular features such as the 2022 trough in OPS 1–945.x. Pandemic-period trends in the present study may therefore reflect denominator effects, altered care-seeking and admission thresholds, psychosocial stress, and changes in documentation or safeguarding workflows, and cannot be read as changes in CPA incidence.

Coding frequencies may also vary between hospitals. In the reimbursement-oriented German coding system, the assignment of non-reimbursement-relevant and medico-legally sensitive codes such as T74.1 may depend on local documentation routines, coding expertise and institutional child-protection structures. At the same time, OPS 1–945.x is reimbursement-relevant, but its financial relevance may vary across hospitals depending on local reimbursement arrangements and implementation within the DRG system. This inter-hospital variability cannot be quantified from aggregated national data, but prior survey evidence of clinician-level variability in reporting thresholds supports plausibility [25, 26].

The predominance of head injuries among hospitalisations with CPA-related coding is consistent with the recognised importance of abusive head trauma [18, 19, 32]. Injury diagnoses alone, however, are not specific for abuse [24]. Psychosocial-context codes showed a different profile: Z61 was mainly associated with psychiatric diagnoses, whereas Z65 was more heterogeneous, likely reflecting differences in clinical settings [14].

Among young children with abuse-relevant fractures and haematomas, T74.1 coding remained uncommon whereas OPS 1–945.x was more frequent and increased, consistent with more children undergoing structured assessment than receiving an explicit CPA diagnosis. The upper-bound proportion of fracture admissions with diagnosis-based CPA-related coding remained low (< 5%), and sensitivity analyses did not materially alter this interpretation. These estimates are below U.S. figures, where approximately 12% of fractures among hospitalised children ≤ 36 months were attributed to abuse [17], with higher proportions in infants. A systematic review by Berthold et al. [24] reports substantially higher abuse probabilities that vary by fracture location and age, with pooled estimates ranging from 17.1–69.4% in infants aged 0–11 months and 4.8–28.5% in children aged 12–23 months. Our exploratory site-specific description showed that femur and humerus fractures had comparatively higher CPA-related coding, and rib fractures, though rare, showed the highest proportions; however, these results are based on aggregated data with cell suppression and should be interpreted descriptively.

A similar pattern was observed for haematomas, where T74.1 remained rare while OPS 1–945.x increased, despite haematomas in young children being recognised clinical warning signs [24]. The low frequency of explicit CPA coding may reflect diagnostic uncertainty or reluctance assigning codes such as T74.1, which explicitly attribute an injury to deliberate maltreatment and may have medico-legal implications. Strengthening assessment pathways and coding guidance may help improve consistency of documentation in this vulnerable age group.

### Limitations

The analysis relies on case-level aggregated data without person-level identifiers. We cannot distinguish incident from repeat presentations, assess overlap between CPA-related codes within the same hospitalisation, or estimate incidence; figures capture only annual inpatient cases carrying relevant codes. Together with cell-suppression rules, this also prevents robust analyses of the highest-risk subgroups and exact bone-specific abuse probabilities. Non-aggregated, longitudinal data will be needed for finer analyses.

The COVID-19 pandemic may have affected the number and case mix of admissions; reporting proportions partly mitigates this, but residual confounding cannot be excluded (discussed above).

The low number of CPA-related codings increases fluctuation in percentage values; averaging across the six-year period reduces this.

As the browser covers inpatient cases only, children assessed for CPA-related concerns in outpatient settings are not captured.

Coding accuracy could not be assessed, as no clinical gold standard is available; German evidence indicates uncertainty and frequent under-recording of maltreatment codes [14, 15], so counts reflect incomplete administrative documentation rather than accurate case counts.

The extended German set has not been validated against clinically adjudicated cases, and several codes (Z61, Z65, Y09.9!) are not specific to physical abuse and may capture neglect, psychosocial adversity or non-abusive injury, so the broader definitions are subject to misclassification in both directions. We therefore report T74.1, broader diagnostic indicators and OPS 1–945.x separately rather than as a single case definition.

Finally, the 0–17 years denominator includes birth-related admissions that dilute population-level proportions; the neonate-free 1 month-17 years and 1 month-5 years strata, the absolute counts and the injury-conditioned proportions are unaffected. As excluding neonates also removes genuine safeguarding activity, the strata should be read as complementary.

## Conclusion

This nationwide analysis of German hospital administrative data (2019–2024) shows that the explicit CPA diagnosis code T74.1 captures only a small fraction of hospitalisations with CPA-related coding, while broader diagnostic indicators and procedural safeguarding coding, particularly OPS 1–945.x, identify substantially more CPA-related documentation and shape observed temporal trends. Among young children with fractures or haematomas—injuries whose probability of being abuse-related depends strongly on age, injury site and clinical context – explicit CPA coding likewise remained rare. As this study evaluates coding rather than clinical performance, this reflects sparse administrative documentation of CPA-related concern, not evidence of missed clinical recognition. Clearer coding guidance, training on administrative documentation, formal validation of broader code sets and European harmonisation of CPA-related coding definitions are needed to improve the surveillance value of hospital administrative data – particularly as the full implementation of the new ICD-11 codes will require several years in most countries.

## Supplementary Information

Below is the link to the electronic supplementary material.

ESM 1 Supplementary Material 1 (DOCX 391 KB)

## Data Availability

Data used in this study were obtained from the publicly accessible InEK Data Browser (Institut für das Entgeltsystem im Krankenhaus, InEK). The data are available at: [https://datenbrowser.inek.org/].

## Abbreviations

CPA: Child Physical Abuse
ICD-10-GM: International Statistical Classification of Diseases and Related Health Problems, 10th Revision, German Modification
OPS: German procedural coding system (Operationen- und Prozedurenschlüssel)
(G-)DRG: (German) Diagnosis Related Group(s)
InEK: Institute for the Hospital Remuneration System (Institut für das Entgeltsystem im Krankenhaus)
KHEntgG: German Hospital Remuneration Act (Krankenhausentgeltgesetz)
PPV: Positive predictive value
DGKiM: German Society for Child Protection in Medicine (Deutsche Gesellschaft für Kinderschutz in der Medizin)
WHO: World Health Organization
