# Supplementary material for: Trends in documenting and coding of child physical abuse in hospitalised children and adolescents in Germany: a nationwide administrative data analysis
Source: Eur J Pediatr. 2026 Aug 3;185(8):636. doi: 10.1007/s00431-026-07300-y (PMC13433376; doi:10.1007/s00431-026-07300-y)
Supplement: Supplementary file 1 [file 431_2026_7300_MOESM1_ESM.docx]

**Table S1** ICD-10-GM codes defining the abuse-relevant injury groups in children aged 0-2 years

| **Code** | **Description** |
| --- | --- |
| S00.05/.45/.55/.7/.85/.95 | Haematomas - superficial injuries of the head, including scalp, ear, oral mucosa, and other/unspecified parts of the head |
| S10.7/.85/.95 | Haematomas - superficial injuries of the neck |
| S20.2/.7 | Haematomas - superficial injuries of the thorax/chest, including contusion of the thorax |
| S30.0/.1/.2/.7 | Haematomas - superficial injuries of the abdomen, lower back, pelvis, and external genitals |
| S02.- | Fractures - fractures of the skull and facial bones |
| S22.3/S22.4 | Fractures - fracture of one rib or multiple ribs |
| S42.- | Fractures - fractures at shoulder and upper-arm level, including clavicle and humerus |
| S52.- | Fractures - fractures of the forearm, including radius and/or ulna |
| S72.- | Fractures - fractures of the femur |
| S82.- | Fractures - fractures of the lower leg, including tibia/fibula and ankle |

**Figure S2** Time-Series-Small-Multiples (1 month-5 years)

T74.1

|  | T74.1 |
| --- | --- |
| 1 | 366 |
| 2 | 405 |
| 3 | 366 |
| 4 | 343 |
| 5 | 311 |
| 6 | 306 |

T71

|  | T71 |
| --- | --- |
| 1 | 32 |
| 2 | 32 |
| 3 | 33 |
| 4 | 31 |
| 5 | 13 |
| 6 | 25 |

T74.8

|  | T74.8 |
| --- | --- |
| 1 | 12 |
| 2 | 16 |
| 3 | 40 |
| 4 | 41 |
| 5 | 26 |
| 6 | 45 |

T74.9

|  | T74.9 |
| --- | --- |
| 1 | 11 |
| 2 | 0 |
| 3 | 14 |
| 4 | 11 |
| 5 | 6 |
| 6 | 17 |

Total diagnostic codes (excl. OPS)

|  | Total diagnostic codes (excl. OPS) |
| --- | --- |
| 1 | 1240 |
| 2 | 1327 |
| 3 | 1340 |
| 4 | 1495 |
| 5 | 1403 |
| 6 | 1501 |

Y09.9!

|  | Y09.9! |
| --- | --- |
| 1 | 40 |
| 2 | 38 |
| 3 | 49 |
| 4 | 33 |
| 5 | 46 |
| 6 | 40 |

Y34.9!

|  | Y34.9! |
| --- | --- |
| 1 | 12 |
| 2 | 10 |
| 3 | 5 |
| 4 | 7 |
| 5 | 11 |
| 6 | 9 |

T76

|  | T76 |
| --- | --- |
| 1 | 12 |
| 2 | 8 |
| 3 | 14 |
| 4 | 13 |
| 5 | 0 |
| 6 | 6 |

Z04.5

|  | Z04.5 |
| --- | --- |
| 1 | 288 |
| 2 | 316 |
| 3 | 335 |
| 4 | 317 |
| 5 | 301 |
| 6 | 391 |

Z61

|  | Z61 |
| --- | --- |
| 1 | 233 |
| 2 | 221 |
| 3 | 210 |
| 4 | 211 |
| 5 | 181 |
| 6 | 214 |

Z65

|  | Z65 |
| --- | --- |
| 1 | 234 |
| 2 | 281 |
| 3 | 274 |
| 4 | 488 |
| 5 | 508 |
| 6 | 448 |

OPS1-945.x

|  | OPS1-945.x |
| --- | --- |
| 1 | 1070 |
| 2 | 1108 |
| 3 | 1147 |
| 4 | 923 |
| 5 | 1166 |
| 6 | 1385 |

Z04.5

|  | Z04.5 |
| --- | --- |
| 1 | 815 |
| 2 | 831 |
| 3 | 895 |
| 4 | 913 |
| 5 | 964 |
| 6 | 1130 |

Z61

|  | Z61 |
| --- | --- |
| 1 | 901 |
| 2 | 842 |
| 3 | 872 |
| 4 | 840 |
| 5 | 833 |
| 6 | 897 |

Z65

|  | Z65 |
| --- | --- |
| 1 | 814 |
| 2 | 1031 |
| 3 | 1069 |
| 4 | 1518 |
| 5 | 1625 |
| 6 | 1639 |

OPS1-945.x

|  | OPS1-945.x |
| --- | --- |
| 1 | 1930 |
| 2 | 2221 |
| 3 | 2251 |
| 4 | 1844 |
| 5 | 2685 |
| 6 | 3147 |

Total diagnostic codes (excl. OPS)

|  | Total diagnostic codes (excl. OPS) |
| --- | --- |
| 1 | 4340 |
| 2 | 4243 |
| 3 | 4577 |
| 4 | 5279 |
| 5 | 5502 |
| 6 | 5884 |

T74.1

|  | T74.1 |
| --- | --- |
| 1 | 555 |
| 2 | 559 |
| 3 | 569 |
| 4 | 552 |
| 5 | 524 |
| 6 | 530 |

T71

|  | T71 |
| --- | --- |
| 1 | 92 |
| 2 | 82 |
| 3 | 92 |
| 4 | 96 |
| 5 | 70 |
| 6 | 94 |

T74.8

|  | T74.8 |
| --- | --- |
| 1 | 26 |
| 2 | 44 |
| 3 | 119 |
| 4 | 218 |
| 5 | 179 |
| 6 | 185 |

T74.9

|  | T74.9 |
| --- | --- |
| 1 | 24 |
| 2 | 11 |
| 3 | 27 |
| 4 | 16 |
| 5 | 14 |
| 6 | 29 |

T76

|  | T76 |
| --- | --- |
| 1 | 61 |
| 2 | 41 |
| 3 | 61 |
| 4 | 46 |
| 5 | 60 |
| 6 | 43 |

Y09.9!

|  | Y09.9! |
| --- | --- |
| 1 | 1024 |
| 2 | 787 |
| 3 | 853 |
| 4 | 1060 |
| 5 | 1207 |
| 6 | 1312 |

Y34.9!

|  | Y34.9! |
| --- | --- |
| 1 | 28 |
| 2 | 15 |
| 3 | 20 |
| 4 | 20 |
| 5 | 26 |
| 6 | 25 |

**Figure S3** Time-Series-Small-Multiples (0-17 years)

**Table S4** Top 3 principal diagnoses

| **Context code** | **0-17 years**  *total cases (%)* | | **1 month-5 years**  *total cases (%)* | |
| --- | --- | --- | --- | --- |
| **T74.1** | S06.5 | 285 (8.7%) | S06.5 | 276 (13.2%) |
|  | S06.0 | 254 (7.7%) | S00.85 | 145 (6.9%) |
|  | S00.85 | 213 (6.5%) | S06.0 | 109 (5.2%) |
| **Z04.5** | S06.0 | 811 (19.1%) | S00.85 | 102 (5.2%) |
|  | S00.85 | 239 (5.6%) | S06.5 | 84 (4.3%) |
|  | S30.1 | 121 (2.9%) | S06.0 | 76 (3.9%) |
| **Y09.9!** | S06.0 | 2,623 (42.0%) | S00.85 | 38 (15.5%) |
|  | S00.85 | 380 (6.1%) | S06.0 | 34 (13.8%) |
|  | S30.1 | 326 (5.2%) |  |  |
| **Z61** | F43.2 | 258 (5.0%) | F83 | 138 (10.9%) |
|  | F93.8 | 225 (4.3%) | F94.1 | 55 (4.3%) |
|  | F83 | 179 (3.5%) | F84.0 | 39 (3.1%) |
| **Z65** | P07.12 | 407 (5.3%) | F83 | 104 (4.7%) |
|  | E10.91 | 152 (2.0%) | J20.9 | 71 (3.2%) |
|  | F83 | 128 (1.7%) | A09.0 | 62 (2.8%) |
|  |  |  |  |  |
| S00-S09 | Injuries to the head | | | |
| S30-S39 | Injuries to the abdomen, lower back, lumbar spine and pelvis | | | |
| F00-F99 | Mental and behavioural disorders | | | |

**Table S5.** Percentages of codes among principal diagnoses of abuse-relevant injuries (haematomas and fractures) in children aged 0-2 years

| **Diagnosis Groups** | **Year** | **Total cases** | **T74.1 n (%)** | **Z04.5 n (%)** | **Y09.9! n (%)** | **T71 n (%)** | **T74.8 n (%)** | **T74.9 n (%)** | **T76 n (%)** | **Z65 n (%)** | **Z61 n (%)** | **OPS 1-945.x n (%)** |
| --- | --- | --- | --- | --- | --- | --- | --- | --- | --- | --- | --- | --- |
| Abuse-relevant **fractures** | 2019 | 3,718 | 53 (1.4%) | 37 (1.0%) | ≤ 4  (≤ 0.1%) | 0 | ≤ 4  (≤ 0.1%) | ≤ 4  (≤ 0.1%) | 0 | ≤ 4  (≤ 0.1%) | ≤ 4  (≤ 0.1%) | 162 (4.4%) |
|  | 2020 | 3,658 | 50 (1.4%) | 31 (0.9%) | ≤ 4  (≤ 0.1%) | 0 | 0 | ≤ 4  (≤ 0.1%) | 0 | 5 (0.1%) | 7 (0.2%) | 147 (4.0%) |
|  | 2021 | 3,369 | 43 (1.3%) | 25 (0.7%) | ≤ 4  (≤ 0.1%) | 0 | ≤ 4  (≤ 0.1%) | 0 | ≤ 4  (≤ 0.1%) | ≤ 4  (≤ 0.1%) | 7 (0.2%) | 155 (4.6%) |
|  | 2022 | 3,174 | 68 (2.1%) | 30 (1.0%) | ≤ 4  (≤ 0.1%) | 0 | 0 | 0 | 0 | 9 (0.3%) | 7 (0.2%) | 149 (4.7%) |
|  | 2023 | 3,213 | 63 (2.0%) | 44 (1.4%) | ≤ 4  (≤ 0.1%) | 0 | 0 | ≤ 4  (≤ 0.1%) | ≤ 4  (≤ 0.1%) | ≤ 4  (≤ 0.1%) | 9 (0.3%) | 186 (5.8%) |
|  | 2024 | 3,175 | 54 (1.7%) | 49 (1.5%) | ≤ 4  (≤ 0.1%) | 0 | ≤ 4  (≤ 0.1%) | 0 | 0 | 9 (0.3%) | 9 (0.3%) | 166 (5.2%) |
| Abuse-relevant **haematomas** | 2019 | 14,674 | 42 (0.3%) | 20 (0.1%) | 7  (< 0.1%) | 0 | 0 | 0 | 0 | ≤ 4  (< 0.1%) | ≤ 4  (< 0.1%) | 65 (0.4%) |
|  | 2020 | 13,795 | 52 (0.4%) | 29 (0.2%) | 10  (0.1%) | 0 | 0 | 0 | 0 | 7  (0.1%) | 7  (0.1%) | 59 (0.4%) |
|  | 2021 | 14,122 | 47 (0.3%) | 24 (0.2%) | 11 (0.1%) | ≤ 4  (< 0.1%) | ≤ 4  (< 0.1%) | 0 | 0 | 5  (< 0.1%) | ≤ 4  (< 0.1%) | 75 (0.5%) |
|  | 2022 | 12,505 | 33 (0.3%) | 23 (0.2%) | 0 | 0 | 0 | 0 | 0 | 0 | 0 | 59 (0.5%) |
|  | 2023 | 13,157 | 43 (0.3%) | 32 (0.2%) | ≤ 4  (< 0.1%) | 0 | 0 | 0 | ≤ 4  (< 0.1%) | ≤ 4  (< 0.1%) | 0 | 77 (0.6%) |
|  | 2024 | 13,342 | 34 (0.3%) | 41 (0.3%) | ≤ 4  (< 0.1%) | 0 | ≤ 4  (< 0.1%) | 0 | 0 | ≤ 4  (< 0.1%) | ≤ 4  (< 0.1%) | 85 (0.6%) |

Note. Cells reported as “≤4” indicate suppressed counts (true value 1–4 cases) that are aggregated. For upper-bound estimates, “≤4” was conservatively set to 4. Because code-specific aggregate counts cannot be linked within individual hospitalisations, the calculation treats diagnosis-code counts as non-overlapping; hospitalisations carrying multiple codes may therefore be counted more than once.

**Table S6.** Sensitivity analysis for suppressed-cell assumptions in diagnosis-based CPA-related coding among predefined fracture and haematoma diagnosis groups, 0-2 years, 2019-2024

| **Injury group** | **Year** | **Admissions** | **Suppressed diagnosis-code cells (n)** | **Sensitivity estimate (suppressed cells = 1), n (%)** | **Upper-bound estimate (suppressed cells = 4), n (%)** |
| --- | --- | --- | --- | --- | --- |
| **Abuse-relevant fractures** | 2019 | 3,718 | 5 | 95 (2.6%) | 110 (3.0%) |
|  | 2020 | 3,658 | 2 | 95 (2.6%) | 101 (2.8%) |
|  | 2021 | 3,369 | 4 | 79 (2.3%) | 91 (2.7%) |
|  | 2022 | 3,174 | 1 | 115 (3.6%) | 118 (3.7%) |
|  | 2023 | 3,213 | 4 | 120 (3.7%) | 132 (4.1%) |
|  | 2024 | 3,175 | 2 | 123 (3.9%) | 129 (4.1%) |
|  | **2019-2024 total** | **20,307** | **18** | **627 (3.1%)** | **681 (3.4%)** |
| **Abuse-relevant haematomas** | 2019 | 14,674 | 2 | 71 (0.5%) | 77 (0.5%) |
|  | 2020 | 13,795 | 0 | 105 (0.8%) | 105 (0.8%) |
|  | 2021 | 14,122 | 3 | 90 (0.6%) | 99 (0.7%) |
|  | 2022 | 12,505 | 0 | 56 (0.5%) | 56 (0.5%) |
|  | 2023 | 13,157 | 3 | 78 (0.6%) | 87 (0.7%) |
|  | 2024 | 13,342 | 4 | 79 (0.6%) | 91 (0.7%) |
|  | **2019-2024 total** | **81,595** | **12** | **479 (0.6%)** | **515 (0.6%)** |

Note. Estimates include ICD-10-GM diagnosis codes only and exclude OPS 1-945.x. Percentages use the number of admissions in each injury group as denominator. Suppressed diagnosis-code cells are cells shown as <=4 in Table S5. Sensitivity estimates assume each suppressed cell equals one case; upper-bound estimates assume each suppressed cell equals four cases. Both calculations treat diagnosis-code counts as non-overlapping because the browser provides separate aggregate counts for each code and does not permit linkage of code combinations within individual hospitalisations. The resulting values are therefore code-based sensitivity estimates rather than exact counts of hospitalisations with at least one CPA-related diagnosis code. Percentages were calculated from summed counts and may therefore differ slightly from sums of rounded code-specific percentages shown in Table S5.

**Table S7.** Exploratory distribution of predefined fracture diagnosis groups by anatomical site among children aged 0-2 years, 2019–2024

| **Fracture site** | **ICD-10-GM principal diagnosis codes** | **Admissions 2019–2024 n (% of site-specific total)** | **T74.1 reportable n (%)** | **Z04.5 reportable n (%)** | **OPS 1-945.x reportable n (%)** |
| --- | --- | --- | --- | --- | --- |
| Skull/facial bones | S02.- | 8,836 (43.5%) | 74 (0.8%) | 49 (0.6%) | 311 (3.5%) |
| Femur | S72.- | 4,223 (20.8%) | 90 (2.1%) | 53 (1.3%) | 314 (7.4%) |
| Radius/ulna | S52.- | 2,525 (12.4%) | 13 (0.5%) | n.r. | 25 (1.0%) |
| Humerus | selected S42.- humerus codes | 2,451 (12.1%) | 77 (3.1%) | 45 (1.8%) | 157 (6.4%) |
| Tibia/fibula | S82.- | 1,706 (8.4%) | 19 (1.1%) | 12 (0.7%) | 67 (3.9%) |
| Clavicle | selected S42.- clavicle codes | 474 (2.3%) | n.r. | n.r. | 5 (1.1%) |
| Ribs | S22.3/S22.4 | 96 (0.5%) | 28 (29.2%) | 5 (5.2%) | 21 (21.9%) |

Note. Percentages in the admissions column refer to the extracted site-specific fracture total (N = 20,311) across 2019–2024. The previously reported broad fracture group comprised 20,307 admissions; the small difference reflects separate aggregate extractions and does not affect interpretation. Code-specific values are sums of individually reportable cells in the InEK browser and should be interpreted as reportable counts/minimum counts. Cells not individually reported may represent zero to four cases because of small-cell suppression and should not be interpreted as true zero counts. n.r. = no annual cell individually reportable. OPS = German procedure classification; CPA = child physical abuse.
